# 21.69–24.36 GHz MEMS Tunable Band-Pass Filter

**DOI:** 10.3390/mi7090149

**Published:** 2016-08-24

**Authors:** Zhongliang Deng, Hao Wei, Xubing Guo

**Affiliations:** School of Electronic Engineering, Beijing University of Posts and Telecommunications, Beijing 100876, China; dengzhl@bupt.edu.cn (Z.D.); gxb@bupt.edu.cn (X.G.)

**Keywords:** microelectromechanical systems (MEMS), tunable band-pass filter, inductively-tuned slow-wave resonator

## Abstract

The K-band microelectromechanical systems (MEMS) tunable band-pass filter, with a wide-frequency tunable range and miniature size, is able to fulfill the requirements of the multiband satellite communication systems. A novel 21.69–24.36 GHz MEMS tunable band-pass filter is designed, analyzed, fabricated and measured. This paper also designs and analyzes an inductively tuned slow-wave resonator, which consists of the MEMS capacitive switch, the MEMS capacitor and the short metal line. The proposed filter has four different work states by changing the capacitance values of the MEMS switches. Measured results demonstrate that, for all four states, the insertion loss is 2.81, 3.27, 3.65 and 4.03 dB at 24.36, 23.2, 22.24 and 21.69 GHz, respectively. The actuation voltage is 0, 20, 16 and 26 V, respectively. The 3 dB bandwidth of the tunable filter is 5.4%, 6.2%, 5.7% and 5.9%, respectively. This study contributes to the design of miniature millimeter tunable filters with a wide-frequency tunable range.

## 1. Introduction

The K-band tunable band-pass filter is one of the essential devices which have been used for modern satellite communication systems and radar systems [1], due to its potential to significantly reduce the overall size and complexity of the systems. Recently, many K-band tunable band-pass filters have been reported; however, the tuning range is limited, for example the tuning range in References [2,3,4,5,6,7] is 1%, 2.5%, 3.1%, 3.8%, 4.5% and 2.3%, respectively.

The waveguide structures of the reported filters include substrate-integrated-waveguide (SIW) [1], cavity [2,3,4], microstrip [5,6] and coplanar waveguide (CPW) [7,8,9,10]. Compared to SIW and cavity technology, microstrip and CPW provide relatively smaller dimensions for the filter. To date, the tune devices for tunable filters contain a radio frequency (RF) MEMS switch, a PIN diode, a stepper motor, a ferroelectric capacitor, a piezoelectric linear actuator and so on. Compared to other technologies, RF MEMS switches have low loss up to the millimeter band and can be easily integrated [11], and they have been widely used in tunable filters with wide tunable ranges [8,9,10,12].

Rebeiz et al. [8] presented an analog tunable band-pass filter fabricated on 500-μm-thick quartz wafer, which tuned over a 14% bandwidth from 18.6 to 21.4 GHz. The filter consisted of 18 MEMS capacitive switches, and the voltage of the switch tuned from 80 to 0 V. The length of the filter was 3.62 mm.

Rebeiz et al. [9] also reported a tunable filter, which was fabricated on 500-μm-thick quartz wafer. This filter utilized low-actuation-voltage MEMS varactors, and its tuning range was from 23.8 GHz (0 V) to 22.6 GHz (15 V). The size of the filter was 6.582 mm × 0.32 mm.

Kim et al. [10] proposed a tunable filter, which was fabricated on 520-μm-thick quartz substrate. This filter tuned from 20.7 to 18 GHz, while the actuation voltages of the MEMS variable capacitors changed from 0 to 35 V. The size of the filter was 2.75 mm × 1 mm.

The methodologies of designing tunable filters in References [8,9,10,12] involved tuning the resonators by changing the loading. In the tunable resonator, the equivalent inductance was built as the MEMS beam, and the variable capacitance was built as the MEMS switch. Generally, the equivalent inductance of the MEMS beam in the K-band tunable filter was small; in References [8,9,10,12] it was ~10, ~10, 15 and 38 pH, respectively. The capacitance ratio of the MEMS switch directly affects the tuning range of the filter. The capacitance ratio of the MEMS switch in References [8,9,10] was only 1.35, 1.3 and 1.31, respectively. Therefore, these filters needed lots of MEMS switches to obtain a wide tuning range.

The decrease of the number of MEMS switches and MEMS capacitors is an effective approach to reduce the complexity and improve the reliability of the device. In the wide tunable filters reported in References [8,9,10,12], the equivalent inductance of the resonator was affected by the MEMS beam structure. Along with the changes of the equivalent inductance, the mechanical property of the MEMS switch will also change. Every change in the system’s mechanics requires repetitions of reliability and qualification tests, making the development very cost-intensive [13].

This paper aims to provide an effective solution for an inductively tuned slow-wave resonator without changing the mechanics of the device, in addition to decreasing the number of MEMS switches and MEMS capacitors. The inductively tuned slow-wave resonator consists of an independent short line, a MEMS capacitor and a MEMS switch which we previously presented in Reference [14]. The relationship between the inductance of the short line and the *S*_21_ parameter of the filter is introduced. To increase the tunable range of the filter, the relationship between the capacitance of the MEMS switch and that of the MEMS capacitor is analyzed. The MEMS capacitor, which is in series with the short line, consists of the metal-air-dielectric-metal (MADM) capacitor and the couple capacitor. Only two low-actuation-voltage MEMS capacitive switches with different sizes, two MEMS capacitors and two short lines with relatively larger inductance are employed in the tunable band-pass filter. An acceptable agreement between the measured and simulated results is achieved. The proposed filter has a tuning range of 11.59% from 21.69 to 24.36 GHz; the 3 dB bandwidth is 5.9%, 5.7%, 6.2% and 5.4% at 21.69, 22.24, 23.2 and 24.36 GHz, respectively. The size of the circuit area is 1.56 mm × 1.46 mm.

## 2. Filter Design

### 2.1. Topology

**Figure 1:**
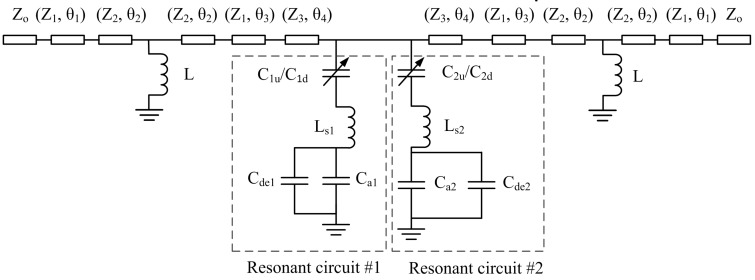
Circuit model of tunable band-pass filter.

The circuit model of the filter is shown in Figure 1. *L* is the inductance of the inverter. Then, (*Z*_1_, θ_1_), (*Z*_2_, θ_2_) and (*Z*_3_, θ_3_) are the characteristic impedances and the electrical lengths of different transmission lines, respectively. Two shunt tunable resonant circuits are formed by (*C_u_*_1_/*C_d_*_1_), (*C_u_*_2_/*C_d_*_2_), *C_a_*_1_, *C_a_*_2_, *C_de_*_1_, *C_de_*_2_, *L_s_*_1_ and *L_s_*_2_. The variable capacitor (*C_u_*_1_/*C_d_*_1_) is built as a MEMS switch, and *C_a_*_1_ is built as a MADM capacitor, so as to (*C_u_*_2_/*C_d_*_2_) and *C_a_*_2_. *L_s_*_1_ and *L_s_*_2_ are built as two independent short metal lines which are the bottom poles for the MEMS switches and MADM capacitors. The short lines cannot connect to the waveguide line, so two couple capacitances (*C_de_*_1_ and *C_de_*_2_) are formed between the terminated short line and the surrounding ground conductor [15]. When the state of the MEMS switches are changed, the resonant circuits will work at different resonant frequencies. The tunable resonant circuit #1 is formed by *C_u_*_1_/*C_d_*_1_, *C_a_*_1_, *C_de_*_1_ and *L_s_*_1_. The resonant frequency *f* is given by

(1) $$f=\frac{1}{2\pi \sqrt{L_{s1}\left(C_{1}||C_{s1}\right)}}$$

where *C*_1_ = *C_a_*_1_ + *C_de_*_1_, and when MEMS switch #1 is in the down state, *C_s_*_1_ = *C_d_*_1_; otherwise, *C_s_*_1_ = *C_u_*_1_. From Equation (1), increasing the value of *L_s_*_1_ can decrease the resonant frequency to further reduce the size of the filter.

(2) $$\frac{C_{1}}{2}<C_{1}||C_{s1}=\frac{C_{1}C_{s1}}{C_{1}+C_{s1}}<C_{1},\;C_{1}<C_{s1}$$

From Equation (2), in order to achieve a relatively wide tunable range for the resonant circuit #1, the dielectric layer is located below MADM capacitor #1 to increase the capacitance *C*_1_. MEMS switch #1 and MADM capacitor #1 share the same electrode. The spring coefficient of switch #1 is less than that of MADM capacitor #1; thus, when MEMS switch #1 is in the down state, MADM capacitor #1 is not pulled down. The design of tunable resonant circuit #2 is the same as tunable resonant circuit #1.

### 2.2. Filter Structure and Tunable Mechanism

**Figure 2:**
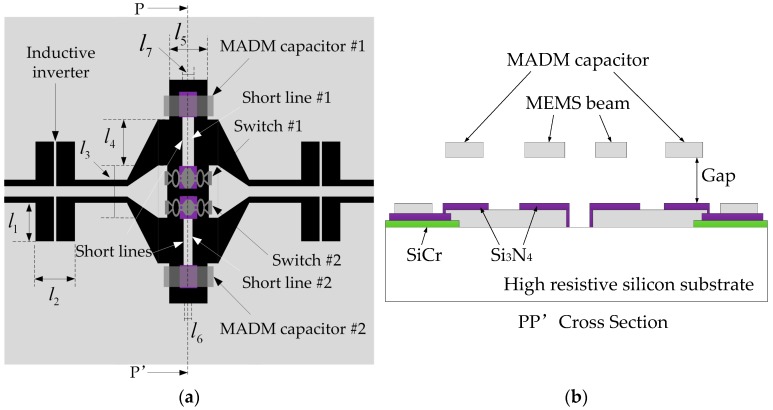
(**a**) The top view of tunable band-pass filter; (**b**) The side view of tunable band-pass filter.

The tunable band-pass filter can be formed by a short-ended half-wave resonator and MEMS switches [7,8,9,10,12]. In order to increase the tunable range of the filter, MEMS switches are in series with the MAM capacitors [12,16]. Figure 2a shows the top view of the filter, and the gray denotes the metal (Au), and the black is the substrate, the MEMS switches are a part of the transmission line. *l*_1_ = 190 μm, *l*_2_ = 220 μm, *l*_3_ = 400 μm, *l*_4_ = 242 μm, *l*_5_ = 204 μm, *l*_6_ = 60 μm, *l*_7_ = 100 μm are the sizes of the proposed filter.

All the resonators are based on 2-μm-thick electroplated gold with dimensions of 63/94/63 μm (G/W/G) on a 400-μm-thick high-resistance silicon (ε_r_ = 11.9, ρ = 4000 Ω·cm and tanδ = 0.0025). The transmission lines use the conductor back coplanar waveguide (CBCPW) structure. All the MEMS switches and MADM capacitors are fabricated using electroplated gold membranes at an average height of 1.1 μm, and the dielectric is Si_3_N_4_ (ε_r_ = 7.6). The high resistance dc bias lines are fabricated using a 50-nm-thick SiCr layer with a resistivity of approximately 1 kΩ/square. The length of the filter is 1560 μm, which is only 0.32 λ_g_ at 24 GHz (λ_g_ = 4930 μm). The side view of the filter is illustrated in Figure 2b. Si_3_N_4_ is located below the MEMS beams. The capacitance of a single MADM capacitor is larger than that of a MAM capacitor, and hence each switch only needs to be in series with one MADM capacitor. The gap between the beam and the dielectric layer is 2 μm. The sizes of the MADM capacitors are both 300 μm × 100 μm, and the sizes of the anchors are both 35 μm × 100 μm. The sizes of short line #1 and short line #2 are 525 μm × 100 μm and 512 μm × 40 μm.

The spring coefficient *k_c_* of the MADM capacitor can be computed by Equation (3) [11].

(3) $$k_{c}=32Ew{\left(\frac{t}{l}\right)}^{3}\frac{1}{8{\left(x/l\right)}^{3}-20{\left(x/l\right)}^{2}+14\left(x/l\right)-1}$$

**Figure 3:**
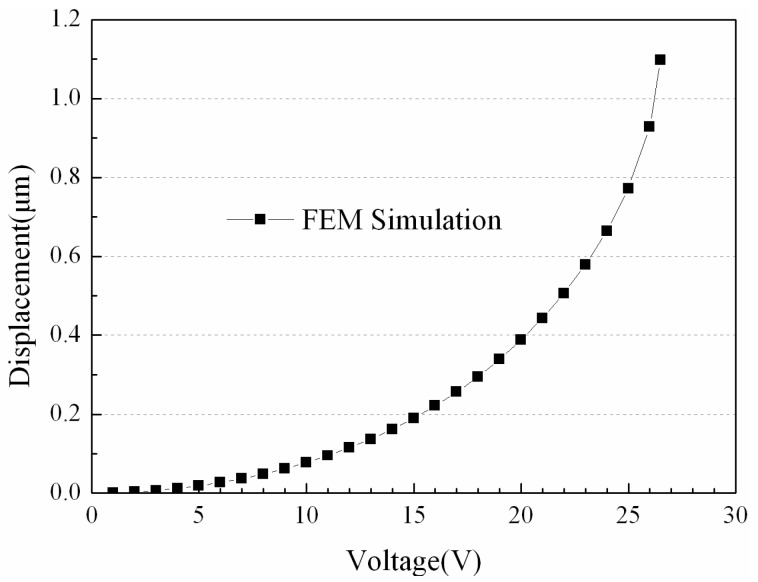
Actuation voltage versus displacements of MEMS beam by FEM simulation.

In Equation (3), the parameters (*t*, *l* and *w*) are the thickness, the length and the width of the beam, respectively. *E* = 78 GPa is the Young’s modulus of Au. For MADM capacitor #1, *x* = 50 μm, and *x* = 20 μm for MADM capacitor #2. The actuation voltage of the MEMS switch can be calculated by Equation (4) [11]. For MADM capacitor #1, the actuation voltage is equal to 27.7129 V from Equation (4). The actuation voltage versus the displacement of the MEMS beam by FEM simulation is shown in Figure 3, and the actuation voltage of MADM capacitor #1 is 26.5 V without residual stress.

(4) $$V_{a}=\sqrt{8k{\left(g+t_{d}/\varepsilon_{r}\right)}^{3}/\left(27\varepsilon_{0}S\right)}$$

where *V_a_* is the actuation voltage, *k* is the spring coefficient, *g* is the distance between the beam and the dielectric layer, *t_d_* is the thickness of Si_3_N_4_, ε_r_ is the relative dielectric constant of Si_3_N_4_, *ε*_0_ is the vacuum dielectric constant, *S* is the size of the center plate.

The capacitance *C* of the MADM capacitor can be given by Equations (5)–(7) [11].

(5) $$C=\frac{\varepsilon_{0}s}{g+\frac{t_{d}}{\varepsilon_{r}}}+C_{f}$$

(6) $$C_{pp}=\frac{\varepsilon_{0}S}{g+\frac{t_{d}}{\varepsilon_{r}}}$$

(7) $$C_{f}\;=0.3-0.4C_{pp}$$

where *C_pp_* is the plate capacitance of the switch, *C_f_* is the edge capacitance of the switch, and the other parameters’ definitions in Equations (5)–(7) are the same as in Equation (4). From Equations (3)–(7) and Figure 3, when the switching voltage of the MADM capacitor is increased, the decrease of *g* results in the increase of the capacitance *C*.

**Figure 4:**
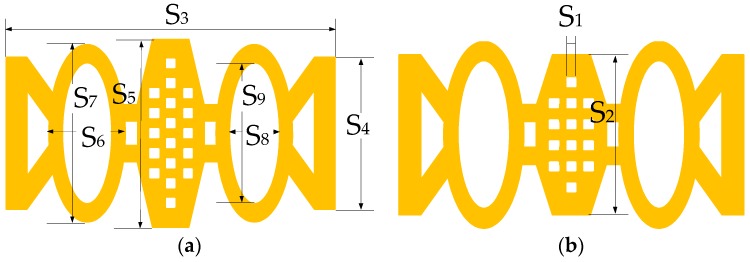
(**a**) Top view of MEMS switch #1; (**b**) Top view of MEMS switch #2.

The top view of MEMS switch #1 and MEMS switch #2 are shown in Figure 4. The values of *S*_1_, *S*_2_, *S*_3_, *S*_4_, *S*_5_, *S*_6_, *S*_7_, *S*_8_ and *S*_9_ are 10, 140, 360, 140, 180, 80, 160, 50 and 120 μm. The size of the center plate of switch #1 is larger than that of switch #2, and the sizes of other parts of switch #1 are the same as those of switch #2. From Equation (4), *V_a_* of switch #1 is larger than that of switch #2.

The state of the MEMS switch is changed when an actuation voltage is applied to the switch. When the filter changes from State-00 (both MEMS switches are in up-state) to State-10, MEMS switch #1 is pulled down. When the filter changes from State-11 (both MEMS switches are in down states) to State-01, MEMS switch #1 is in the up state.

### 2.3. Parameters Calculation and Simulations

Figure 1 and Figure 2 illustrate that (*C_u_*_1_, *C_d_*_1_), (*C_u_*_2_, *C_d_*_2_), (*C_a_*_1_, *C_a_*_2_), (*C_de_*_1_, *C_de_*_2_) and *L* are the up-state capacitance and down-state capacitance of switch #1, the up-state capacitance and down-state capacitance of switch #2, the capacitances of MADM capacitor #1 and MADM capacitor #2, the equivalent capacitances of the open circuit between the terminated short lines and the surrounding ground, and the inductance of the inverter, respectively; (*L_s_*_1_, *L_s_*_2_) are the equivalent inductances of short line #1 and short line #2, and those widths are (*l*_7_, *l*_6_). Further, (*Z*_1_, θ_1_), (*Z*_2_, θ_2_) and (*Z*_3_, θ_3_) are the characteristic impedances and the electrical lengths of the transmission lines with different sizes, respectively.

The parameters (*C_u_*_1_, *C_u_*_2_, *C_a_*_1_, *C_a_*_2_, *L*, *Z*_1_, *Z*_2_, *Z*_3_, θ_1_, θ_2_, θ_3_ and θ_4_) of the circuit are obtained by HFSS and ADS software, where *C_u_*_1_ = 59.4 fF, *C_u_*_2_ = 44.2 fF, *C_a_*_1_ = 54.5 fF, *C_a_*_2_ = 35 fF, *L* = 90 pH, *Z*_1_ = 49.6786 Ω, *Z*_2_ = 69.6541 Ω, *Z*_3_ = 40.3103 Ω. θ_1_ = 43.7397, θ_2_ = 6.22315, θ_3_ = 19.7436 and θ_4_ = 21.2671 at 20 GHz.

*C_d_*_1_ = 4.54 pF and *C_d_*_2_ = 2.78 pF are calculated by the plate capacitance model [11]. *L_s_*_1_ = 65.8 pH is calculated by Equations (8) [17], and μ is the permeability of vacuum; similarly, *L_s_*_2_ = 96.6 pH.

(8) $$L_{s1}=\frac{\mu l_{4}}{2\pi }\left\{\frac{l_{7}}{4l_{4}}-\sqrt{1+{\left(\frac{l_{7}}{4l_{4}}\right)}^{2}}+ln\left(1+\sqrt{1+{\left(\frac{l_{7}}{4l_{4}}\right)}^{2}}/\frac{l_{7}}{4l_{4}}\right)\right\}$$

**Figure 5:**
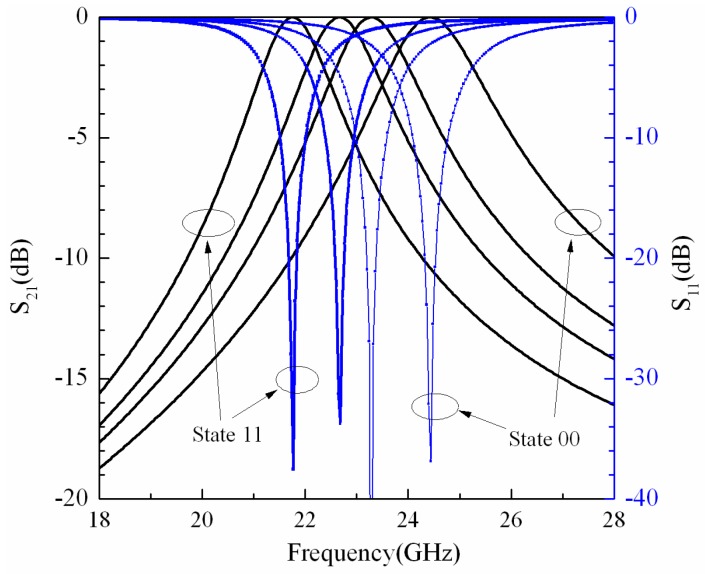
Return loss and insertion loss of simulation results.

*C_de_*_1_ = 9.2 fF is approximately computed by Equation (9) [18], where *h* is the thickness of the substrate, (β_1_, *Z*_o1_, ε_r_, ε_eff1_) are the phase constant, the characteristic impedance, the substrate dielectric constant, the equivalent dielectric constant of the CBCPW transmission line (G/S/G = 52/100/52 μm), respectively; and they are given by Equations (10)–(12) [19], where *c* is the velocity of light in free space, ω is the angular frequency; *K*(*k*), *K*(*k*′), *K*(*k*_1_) and *K*(*k*_1_′) are the complete elliptic integrals; similarly, *C_de_*_2_ = 7.2 fF.

(9) $$C_{de1}=\tan\left(\beta_{1}l_{5}/4\right)/\omega Z_{o1}$$

(10) $$\beta_{1}=\omega \sqrt{\varepsilon_{\text{eff}1}}/c$$

(11) $$\varepsilon_{\text{eff}1}=\frac{1+\varepsilon_{r}\left[K\left(k^{\prime }\right)/K\left(k\right)\right]\left[K\left(k_{1}\right)/K\left(k_{1}^{\prime }\right)\right]}{1+\left[K\left(k^{\prime }\right)/K\left(k\right)\right]\left[K\left(k_{1}\right)/K\left(k_{1}^{\prime }\right)\right]}$$

(12) $$Z_{o1}=\frac{60\pi }{\sqrt{\varepsilon_{\text{eff}1}}}\frac{1}{K\left(k\right)/K\left(k^{\prime }\right)+K\left(k_{1}^{\prime }\right)/K\left(k_{1}\right)}$$

where *k* = *l_7_*/*l_5_*, *k*_1_ = tan*h*(π*l*_7_/(4*h*))/tan*h*(π*l*_5_/(4*h*)), *k*′ = (1 − *k*^2^)^(1/2)^, *k*_1_′ = (1 − *k*_1_^2^)^(1/2)^. Figure 5 shows the return loss and insertion loss of the simulation results. *S* (*S*_11_ and *S*_21_) parameters and the ABCD matrix are given by [20]

(13) $$\left[\begin{array}{cc} A & B\\ C & D \end{array}\right]=\left[M_{1}\right]\left[M_{2}\right]\left[M_{3}\right]\left[M_{2}\right]\left[M_{4}\right]\left[M_{5}\right]\left[M_{6}\right]\left[M_{7}\right]\left[M_{5}\right]\left[M_{4}\right]\left[M_{2}\right]\left[M_{3}\right]\left[M_{2}\right]\left[M_{1}\right]$$

(14) $$S_{11}=\frac{A+B/Z_{O}-C\times Z_{O}-D}{A+B/Z_{O}+C\times Z_{O}+D}\;S_{21}=\frac{2}{A+B/Z_{O}+C\times Z_{O}+D}$$

where

$$\left[M_{1}\right]=\left[\begin{array}{cc} \text{cos}\theta_{1} & Z_{1}\text{tan}\theta_{1}\\ j\text{sin}\theta_{1}/Z_{1} & \text{cos}\theta_{1} \end{array}\right]\;\left[M_{2}\right]=\left[\begin{array}{cc} \text{cos}\theta_{2} & Z_{2}\text{tan}\theta_{2}\\ j\text{sin}\theta_{2}/Z_{2} & \text{cos}\theta_{2} \end{array}\right]$$

$$\left[M_{3}\right]=\left[\begin{array}{cc} 1 & 0\\ {\left(j\omega L\right)}^{-1} & 1 \end{array}\right]\;\left[M_{4}\right]=\left[\begin{array}{cc} \text{cos}\theta_{3} & Z_{1}\text{tan}\theta_{3}\\ j\text{sin}\theta_{3}/Z_{1} & \text{cos}\theta_{3} \end{array}\right]$$

$$\left[M_{5}\right]=\left[\begin{array}{cc} \text{cos}\theta_{4} & Z_{3}\text{tan}\theta_{4}\\ j\text{sin}\theta_{4}/Z_{3} & \text{cos}\theta_{4} \end{array}\right]\;\left[M_{6}\right]=\left[\begin{array}{cc} 1 & 0\\ Y_{s1} & 1 \end{array}\right]$$

$$\left[M_{7}\right]=\left[\begin{array}{cc} 1 & 0\\ Y_{s2} & 1 \end{array}\right]$$

$$Y_{s1}={\left\{1/\left(j\omega \left(\left(C_{a1}+C_{de1}\right)||C_{s1}\right)+j\omega L_{s1}\right)\right\}}^{-1}$$

$$Y_{s2}={\left\{1/\left(j\omega \left(\left(C_{a2}+C_{de2}\right)||C_{s2}\right)+j\omega L_{s2}\right)\right\}}^{-1}$$

**Figure 6:**
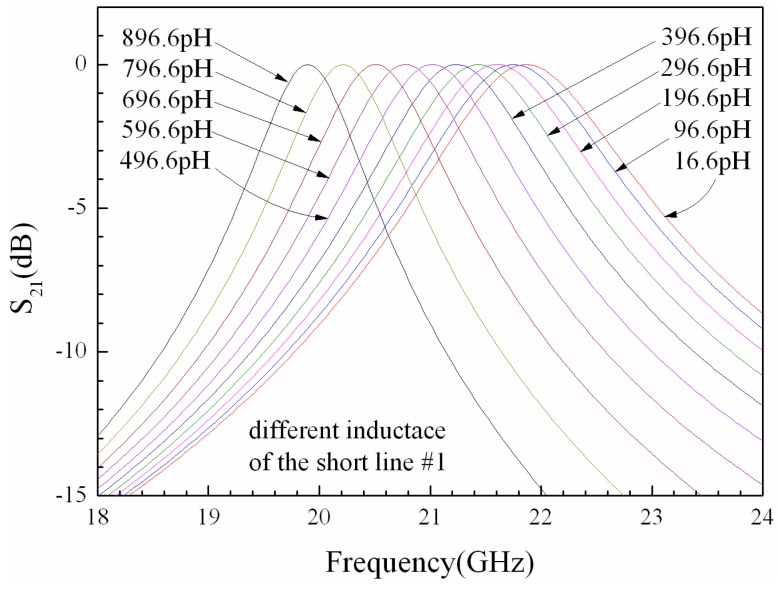
The inductance of short line #1 versus *S*_21_ parameter of the filter.

The resonant frequency of the filter can be tuned by changing the inductance of the short lines. We carry on the simulation by varying the inductance of short line #1 while keeping all the others at their default values. Figure 6 shows the relationship between the inductance of short line #1 and the *S*_21_ parameter of the filter using ADS software. When the inductance of short line #1 is increased, the center frequency of the filter is decreased, and the bandwidth is reduced.

### 2.4. Fabrication

**Figure 7:**
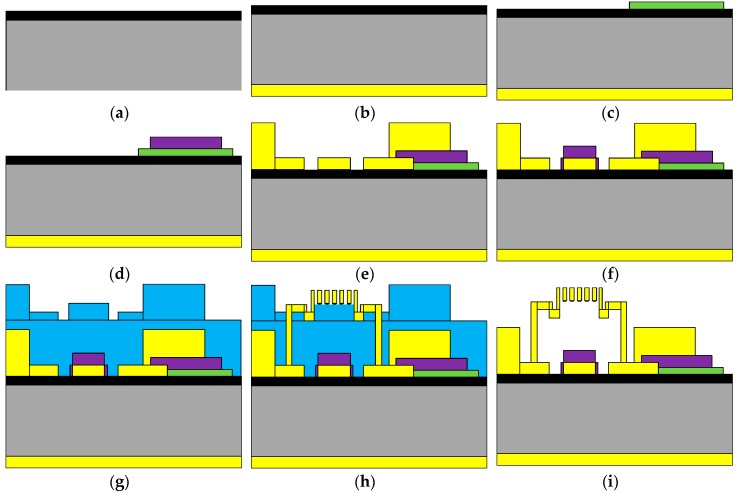

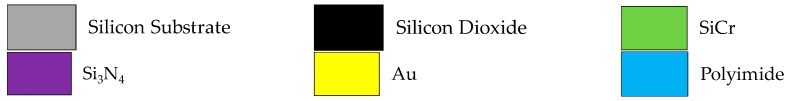
(**a**) After oxidation; (**b**) After sputtering of Au; (**c**) After patterning of high-resistance dc bias line; (**d**) After patterning of Si_3_N_4_; (**e**) After patterning of CPW lines; (**f**) After patterning of Si_3_N_4_; (**g**) Spinning of polyimide to form sacrificial layer; (**h**) Patterning of gold layer to form MEMS beams and anchors; (**i**) Removal of sacrificial layer.

**Figure 8:**
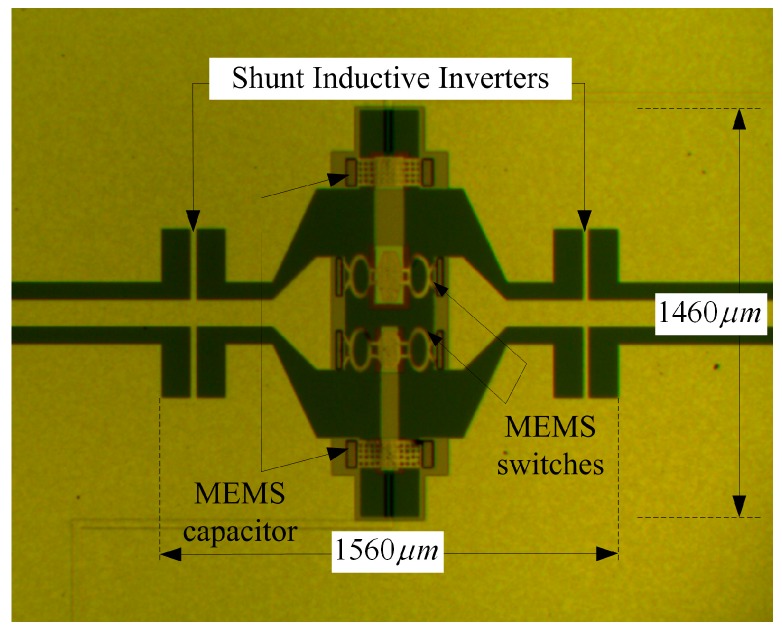
Micrograph of fabricated tunable band-pass filter.

The fabrication process of the device is shown in Figure 7. The device is fabricated on 400-μm-thick high resistance silicon. Further, 0.2-μm-thick Au is located at the bottom of the substrate, and 80-nm-thick silicon dioxide is grown on the substrate by means of thermal oxidation. The high resistance DC bias lines are sputtered and patterned, and 0.16-μm-thick Si_3_N_4_ is deposited on top of the bias lines. The transmission line consists of a 0.2-μm-thick Au center conductor and 2-μm-thick Au ground planes. The bottom electrode is covered with a 0.16-μm-thick Si_3_N_4_ layer, which is deposited using the plasma-enhanced chemical vapor deposition (PECVD) for DC isolation. A 2-μm-thick polyimide is as the sacrificial layer after the thermal curing process. The anchors and the beams are formed by electroplating. The thickness of the MEMS beam is about 1.1 μm. To avoid collapsing the membrane during drying, the structure can be released using a critical drying technology [21,22]. Figure 8 shows the micrograph of the fabricated tunable band-pass filter. One end of the high-resistance line is connected to the electrode, and the other end is connected to the short line.

## 3. Measured Results and Discussion

The device is measured in an unpackaged and standard laboratory environment. The Vector Network Analyzer (R&S ZVA50, Rohde & Schwarz, Munich, Germany) is used to measure the *S* (*S*_11_ and *S*_21_) parameters of the device. Two gold ACP-A-GSG-150 probes (Cascade Microtech, Beaverton, OR, USA) are used to contact the two ends of the device, and the device is placed on a probe table (Cascade Summit 11000B-M, Cascade Microtech, Beaverton, OR, USA). The sweep frequency is from 0.1 to 40 GHz.

**Figure 9:**
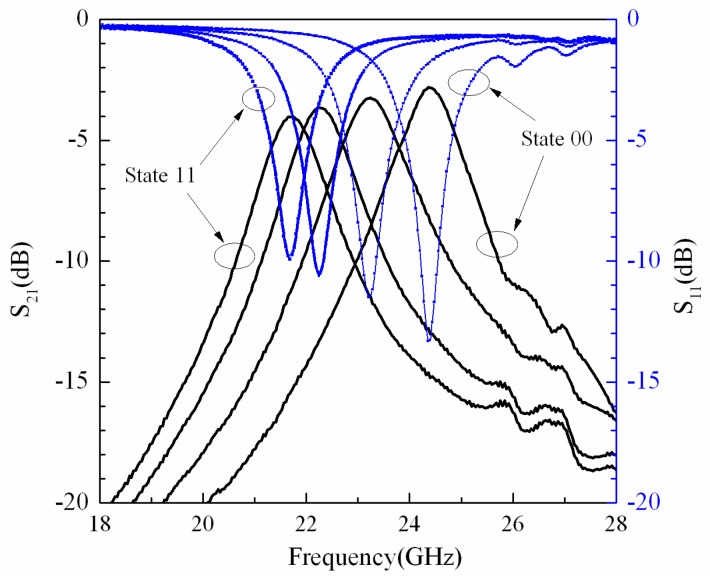
Return loss and insertion loss of measured results of tunable band-pass filter.

Figure 9 shows the return loss and the insertion loss of the measured results. The tunable MEMS filter works at the center frequency of 24.36 GHz with the insertion loss of 2.81 dB when both the MEMS switches are in the up state, and the 3 dB bandwidth is 5.4% (1.3167 GHz); meanwhile, *S*_11_ is −13.31 dB. When MEMS switch #2 is pulled down by loading the actuation voltage of 20 V, the center frequency of the filter is 23.2 GHz, the insertion loss and the return loss are 3.27 dB and −11.5 dB, respectively; the 3 dB bandwidth is 1.4364 GHz. Releasing MEMS switch #2, and when MEMS switch #1 is pulled down by the actuation voltage of 16 V, the center frequency of the filter is 22.24 GHz, *S*_21_ and *S*_11_ are 3.65 dB and −10.61 dB, respectively; the 3 dB bandwidth is 1.2768 GHz. When the actuation voltage is increased up to 26 V, the center frequency of the filter is changed from 22.24 to 21.69 GHz, the insertion loss is 4.03 dB, the return loss is −9.94 dB, and the 3 dB bandwidth is 1.2768 GHz.

**Figure 10:**
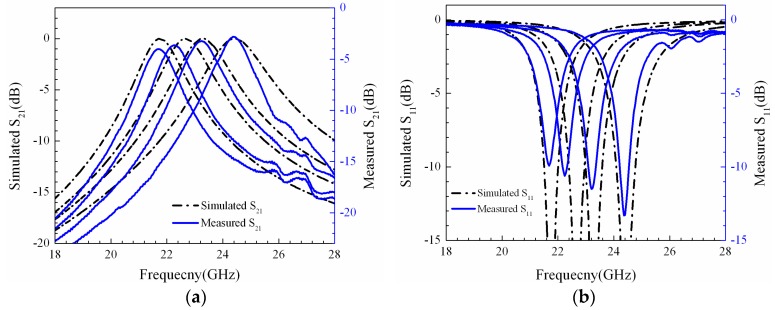
(**a**) *S*_21_ of measured results and simulation results; (**b**) *S*_11_ of measured results and simulation results.

As shown in Figure 10, the blue lines denote the measured results, and the dashed lines represent the simulation results. The minimum measured *S*_21_ of the filter is 2.81 dB, and the circuit simulated *S*_21_ of the filter is close to 0. In order to observe the degree of fitting of the center frequencies and bandwidths between the measured results and simulation results, we keep the −3 dB axis of the measured *S*_21_ aligned with the 0 dB axis of the simulated *S*_21_. For State-11, State-01 and State-00 of the filter, the center frequencies of the measured results are essentially in agreement with the simulation results. There are errors between the actual parameters and the designed parameters of the device, for instance the size error of the metal line, the gap error between the MEMS beam and the dielectric, and the thickness error of the dielectric. For State-10 of the filter, the measured center frequency is less than the simulation result. The reason is that when the actuate voltage is applied to short line #1, MEMS switch #1 is pulled down; meanwhile, the height of MADM capacitor #1 is decreased, which results in the increase of the capacitances of the MADM capacitors. The fabricated widths of the metal lines are ~3 μm larger than the designed values. The errors cause the decrease of the inductances of the investors, so the measured bandwidth is less than the simulation results. The errors between the fabricated sizes of the G/S/G transmission line and the designed parameters are ~3 μm after electroplating, so the absolute values of the measured *S*_11_ are less than the simulation results.

## 4. Conclusions

A miniature tunable MEMS band-pass filter with a relatively wide tunable range and bandwidth is proposed. The MEMS switch is in series with the MEMS capacitor by the short metal line, which has a relatively larger inductance. The filter has a wide tuning range of 11.59% (21.69–24.36 GHz) while the voltage changes from 26 to 0 V, and the 3 dB bandwidth is 5.9%, 5.7%, 6.2% and 5.4% at 21.69, 22.24, 23.2 and 24.36 GHz, respectively. Meanwhile, the insertion loss is 2.81, 3.27, 3.65 and 4.03 dB, individually. The circuit area is only 1.56 mm × 1.46 mm. Such a K-band miniature tunable filter can work in different states, and so it has found wide applications in many fields such as space and 5G communication systems.
